#  Busulfan induced azoospermia: Stereological evaluation of testes in rat

**Published:** 2015-12-15

**Authors:** Mohadeseh Panahi, Saeideh Keshavarz, Farhad Rahmanifar, Amin Tamadon, Davood Mehrabani, Negar Karimaghai, Masood Sepehrimanesh, Heydar Aqababa

**Affiliations:** 1 *Stem Cell and Transgenic Technology Research Center, Shiraz University of Medical Sciences, Shiraz, Iran;*; 2 *Department of Biology, Islamic Azad University, Arsanjan Branch, Arsanjan, Iran;*; 3 *Department of Basic Sciences, School of Veterinary Medicine, Shiraz University, Shiraz, Iran;*; 4 *Gastroenterohepatology Research Center, Shiraz University of Medical Sciences, Shiraz, Iran.*

**Keywords:** Busulfan, Infertility, Rat, Stereology, Testes

## Abstract

The aim of the present study was stereological evaluation of testes of azoospermic animal model using busulfan in rat. Three groups of male adult rats were used in this study. The first group was injected by single dose of busulfan (10 mg kg^-1^) and their testes were removed on day 35 post injection. The second group received double doses of busulfan with 21 days interval and their testes were removed on day 35 after the second injection. The testes of the third group were removed without busulfan therapy. In 10 circular transverse sections of tubules stained with hematoxylin-eosin, stereological parameters were measured. The testes were rated for its spermatogenic potential on a modified spermatogenic scale of 0 to 6. Cellular (germinal epithelium) diameter and area of the seminiferous tubules, total diameter and cross sectional area of the tubules of the seminiferous tubules in rats that received double doses of busulfan were less than the rats in single dose of busulfan and control groups (*p *< 0.05). Spermatogenesis index of seminiferous tubules in rats receiving two doses of busulfan was less than the rats received one dose of busulfan (*p* < 0.001) and the index of both treatment groups were less than the control group (*p* < 0.001). In conclusion, two doses of busulfan injection with 21 days interval produced an appropriate experimental model of induced azoospermia with comparable stereological indices of seminiferous tubules in rat.

## Introduction

Busulfan, 1,4-bis [methanesulfonyl-y] butane, is a chemotherapeutic agent that most often is used as low dose in a long time manner to treat chronic myeloid leukaemia.^1^ Also, it has been given in higher doses before a bone marrow or stem cell transplant for other types of cancer^2^^,^^3^ and hematological stem cell transplant for nearly 20 years.^4^ Busulfan is an alkylating agent, which inhibits cell division by sticking to one of the DNA strands.^5^ Therefore, organs, tissues and cells with high division activities such as testes and germ cells are more susceptible to busulfan side effects. Spermatogenesis involves the passage of diploid germ cells through the reductive divisions of meiosis in order to generate round haploid spermatids.^6^ It is clear that cytotoxic therapy such as certain chemotherapy drugs influences spermatogenesis at least temporarily and in some cases permanently. Busulfan-treated mice exhibited a marked increase in apoptosis and a decrease in testis weight.^7^ Also, single doses of busulfan can permanently sterilize mice at non-lethal doses and cause long-term morphological damage to sperm produced by surviving spermatogonia.^8^ On the other hand, in molecular mechanism, it has been demonstrated that increased depletion of male germ cells in the busulfan-treated mice is mediated by loss of c-kit/SCF signaling, not by p53- or *Fas/FasL*-dependent mechanisms.^7^

The number of malignant diseases is increased worldwide and people diagnosed with cancer currently have a high chance of survival as a result of new treatment protocols such as surgery, combination chemotherapy and radiation therapy.^9^ Although, malignant diseases might influence gonadal function through hormonal changes, however, the negative effects of cytotoxic drugs on spermatogenesis are more important.^10^ The number of reports about busulfan side effects on testicular architecture and fertility indices in animal model are scarce, therefore, the aims of this study were to evaluate the stereological changes of testicular tissue in response to single and two dose of busulfan injection in male rat and comparing them together and with normal control. 

## Materials and Methods

**Animals. **Fifteen male adult Sprague-Dawley rats (200 ± 10 g) were housed in cages under controlled temperature (22.0 ± 2.0 ˚C) and lighting (14 light/10 dark; starting at 07:00 through 21:00) with free access to pellet diet and water in Laboratory Animal Center of Shiraz University of Medical Sciences, Shiraz, Iran. The animals were treated humanely and in compliance with the recommendations of the Institutional Animal Care Committee.

**Busulfan therapy and sampling. **The animals were randomly assigned into three groups. The first group of rats were injected a single dose^11^ (10 mg kg^-1^, intra peritoneal) of busulfan (Busilvex^®^; Pierre Fabre Medicament, Boulogne, France) and their testes were removed on day 35 post injection. The second group of animals received double doses of busulfan with 21 days interval^12^ and their testes were removed on day 35 after the second injection, and the third group of animals was selected as control and their testes were removed. Spermatogenesis in rats takes 56 days (21+35 days),^7^ therefore, this time was selected for complete azoospermia. On days of sampling animals were euthanized with ether and their testes were collected and fixed in a 10% formalin buffer solution. After fixation, segments were embedded in paraffin, and histological sections were made from each block. The 5 µm thickness sections were stained with hematoxylin-eosin.

Sections were visualized and photographed on light microscope (Model CX21; Olympus, Tokyo, Japan) equipped with an adjusted digital camera (AM423U Eyepiece Camera; Dino-Eye, San-Chung, Taiwan).

**Fig. 1:**
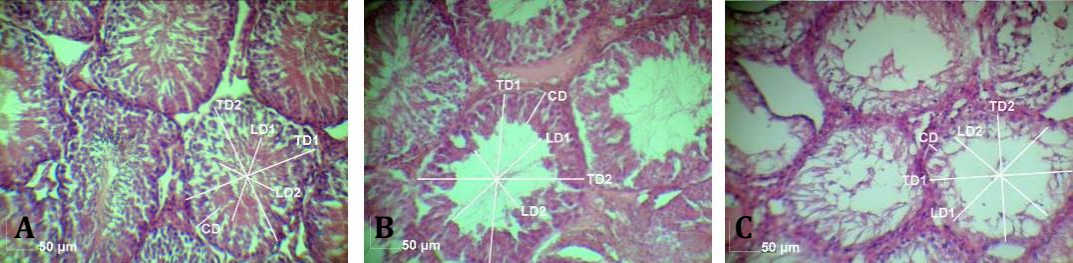
Seminiferous tubules in rats with **A) **normal testes, **B) **single dose- (hypospermatogenesis) and **C) **double dose (Sertoli cell only) of busulfan. TD1 and 2: total diameter, LD1 and 2: lumen diameter, CD: Cellular diameter (Hematoxylin and eosin

**Fig. 2:**
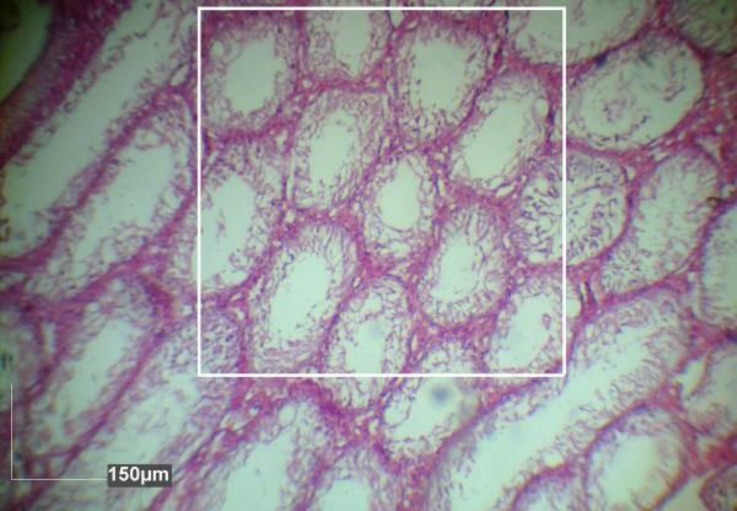
The number of profiles of seminiferous tubules in rats with double doses busulfan injected testes per unit area was determined using the unbiased counting frame (0.5 × 0.5 mm^2^), (Hematoxylin and eosin

**Stereological analysis. **Five vertical sections from the polar and the equatorial regions were sampled in each testis. In one cross-section per animal, all tubules were evaluated for presence of spermatogonia, spermatocytes and spermatids. Inner, outer and total diameters were measured in 10 circular transverse sections of semini-ferous tubules from different regions of the testis (Fig. 1). Using diameter data, areas of the cellular (germinal epithelium) and luminal regions and cross sectional area of the tubules were calculated. Also, the number of late spermatids were counted and then volume density and absolute volume of the seminiferous epithelium and lumen, number of profiles of seminiferous tubules per unit area of testis were calculated (Fig. 2), and numerical density of the seminiferous tubules using a systematic random scheme were measured.^13^^,^^14^ Diameters and number of tubules were measured on transverse sections using Dinocapture 2.0 software (Dino-Eye, San-Chung, Taiwan).

The mean seminiferous tubule diameter (D) was derived by taking the average of two diameters, D_1_ and D_2_ at right angles. Cross-sectional area (*Ac*) of the seminiferous tubules was determined using the following equation:

Ac =π D24

where, *π* is equivalent to 3.142 and *D* is the mean diameter of seminiferous tubules. The number of profiles of seminiferous tubules per unit area (N_A_) was determined using the unbiased counting frame.^15^ Numerical density (N_v_) of seminiferous tubules was the number of profiles per unit volume and it was measured using the modified Floderus equation:^16^

$Nv=\frac{N_{A}}{D+T}$

where, *N*_A_ is the number of profiles per unit area, *D* is the mean diameter of the seminiferous tubule and *T* the average thickness of the section.

A testis was rated for its spermatogenic potential (modified spermatogenic index) on a scale of 0 to 6.^17^ The index was based on the appearance of the spermatogenic cells throughout the testis and included number of cell layers, types of cells and the presence of late spermatids in the seminiferous tubules. The index and criteria were as follow: 0 = no spermatogenic cells; 1 = only spermato-gonia present; 2 = spermatogonia and spermatocytes present; 3 = spermatogonia, spermatocytes and round (early) spermatids present with < 25 late spermatids per tubule; 4 = spermatogonia, spermatocytes, and round spermatids present, and up to 50 late spermatids per tubule; 5 = all cell types present and 50 to 100 late spermatids per tubule; 6 = all cell types present and > 100 late spermatids per tubule.

**Statistical analysis. **Means and standard error (SE) of the data of stereological indices of seminiferous tubules were subjected to Kolmogorov-Smirnov test of normality and analyzed by one-way ANOVA using SPSS (version 11.5; SPSS Inc., Chicago, USA), and post-hoc test was performed by LSD test. The spermatogenesis index of seminiferous tubules was compared using Mann-Whitney U test. A *p*-value of less than 0.05 was considered to be statistically significant. Group means and their standard error were reported in the text and graphs using GraphPad Prism (version 5.01; GraphPad software Inc., San Diego, USA).

## Results

Control group presented tubules with thin basement membrane and tunica propria as well as normal germinal epithelium showing orderly progression from spermato-gonia to spermatocytes with groups of spermatids and mature spermatozoa (Fig. 1A). Sertoli cells were compressed between the germinal cells and were not easily seen. In single dose of busulfan group, hypospermatogenesis was observed. The cellularity of germinal epithelium was reduced at all stages, the numbers of all types of germ cells (spermatogonia, spermatocytes, and spermatids) were reduced but had normal Leydig cells (Fig. 1B). Moreover, in some tubules maturation was arrested and histopatho logical description of the interruption of normal germ cell maturation at the level of a specific cell type leading spermatogonia to spermatids (spermatogonia arrest, spermatocyte arrest, or spermatid arrest). In double dose of busulfan group, only Sertoli cell and complete germ cell aplasia were present. The tunica propria and basement membranes were not thickened appreciably, and the tubules were normal or slightly decreased in diameter, and contained only Sertoli cells but no other cells were involved in spermatogenesis. The interstitium contained normal numbers of Leydig cells (Fig. 1C).

**Fig. 3:**
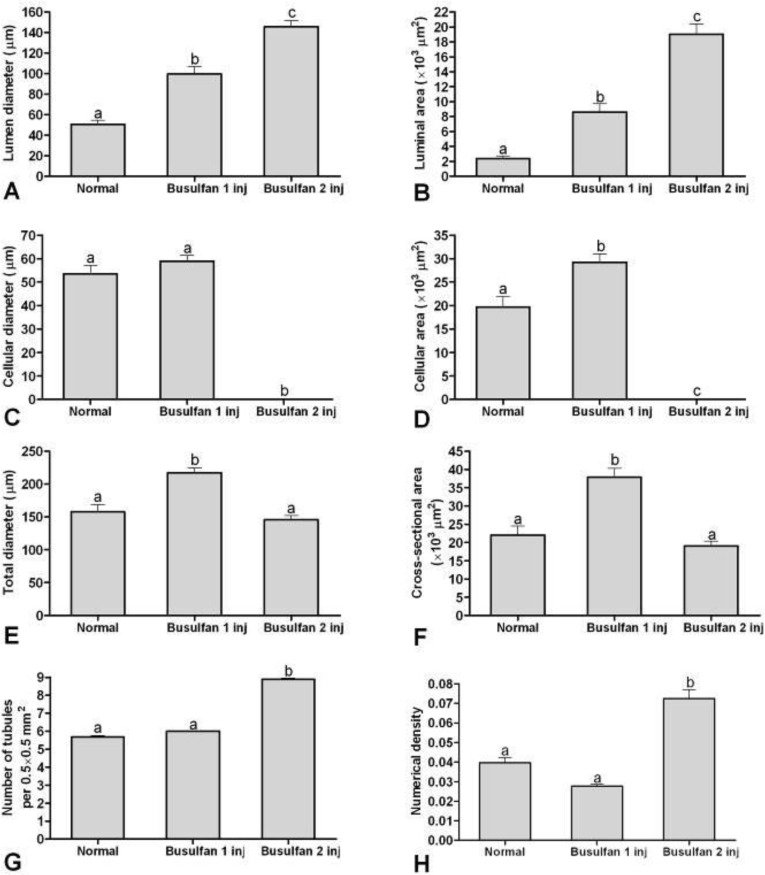
Mean and standard error of stereological indices of seminiferous tubules in rats with normal, single dose and double doses busulfan injected testes. **A)** Lumen diameter (μm), **B)** Luminal area (μm^2^), **C)** Cellular diameter (μm), **D)** Cellular area (μm^2^), **E)** Total diameters (μm), **F)** Cross sectional area of the tubule (μm^2^), **G)** Number of seminiferous tubules per unit area of testis, **H)** Numerical density of the seminiferous tubules.

^abc^ Different superscript letters show significant differences between groups (*p* < 0.05).

In stereological indices, lumen diameter and luminal area of the seminiferous tubules in rats that received two doses of busulfan were more than the rats in one dose of busulfan injected (*p* < 0.001) and those of both treatment groups were more than control groups (*p* < 0.05; Figs. 1, 3A and 3B). Cellular diameter and cellular area of the seminiferous tubules in rats that received two doses of busulfan were less than the rats in one dose of busulfan injected and control groups (*p* < 0.001; Figs. 3C and 3D). 

The mean of cellular area of the rat that received one dose of busulfan was more than control group (*p* < 0.001), an increase was seen in cellular diameter and cellular area of the one dose busulfan injected rats while detachment and scattering of the cells in the cellular layer of seminiferous tubules were increased after the first injection (Fig. 1B).

Total diameter and cross sectional area of the semini-ferous tubules in rats that received one doses of busulfan was more than the rats in two dose of busulfan injected (*p* < 0.001) and control groups (*p* < 0.001; Figs. 3E and 3F).

Number of seminiferous tubules per unit area of testis and numerical density of the seminiferous tubules in rats that received two doses of busulfan was more than the rats received one dose of busulfan (*p* < 0.001) and the control group (*p* < 0.001; Figs. 3G and 3H).

**Fig. 4:**
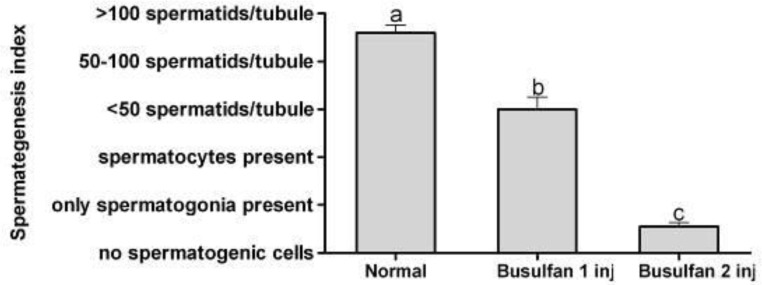
Mean and standard error of spermatogenesis index of seminiferous tubules in rats with normal, single dose and double doses busulfan injected testes

^abc^ Different superscript letters show significant differences between groups (*p* < 0.05).

Spermatogenesis index of seminiferous tubules in rats receiving two doses of busulfan was less than the rats received one dose of busulfan (*p* < 0.001) and the index of both treatment groups were less than the control group (*p* < 0.001; Fig. 4).

## Discussion

In the present study, alterations of rat testicular histopathology and spermatogenesis potency in response to one and two injections of busulfan were evaluated. Our results demonstrated that two doses of busulfan injections with 21 days interval produced an appropriate animal model of induced azoospermia with comparable stereo-logical indices of seminiferous tubules 35 days after the last injection in Sprague-Dawley rats. Busulfan is a potent agent that preferentially kills spermatogonial stem cells of several species, however, it has no effects on DNA synthesis. However, it inhibits the next mitosis when it intoxicates the cells in the G1 phase.^18^^,^^19^ In the previous study, the morpho-logical behavior of spermatogonia following recovery from two doses of busulfan treatment in rat was reported. Twenty days after the second intraperitoneal injection of busulfan, the testes lost most of their spermatogenic cells and there were fewer dispersed singly surviving spermato-gonia.^19^ Similarly, the effects of busulfan administration on testicular tissues of rat pubs were evaluated by Krause.^21^ Busulfan in 10 mg kg^-1^ was administered on various days of gestation to Wistar rats and the testes of the male pubs of these litters were examined histologically.^21^ Depending on the time of application of busulfan, the cells of spermio-genesis were diminished and the meiosis was delayed, however, the Leydig cells were unchanged. In the second part of that study, the weight of the seminal vesicles, luteinizing hormone and follicle-stimulating hormone in the serum showed no differences versus the control group.^21^

Our results were in agreement with results which obtained by all above mentioned studies^18^^-^^21^ and showed that two dose of busulfan injections in male rat could induce azoospermia.

On the other hand, there are several approaches for malignancy therapy such as chemical and radiotherapy. It has been shown that fractionated irradiation results in depletion of endogenous spermatogenesis similar to using busulfan in doses of 50 to 55 mg kg^-1^.^22^ Although, this phenomenon can direct researchers to this fact that locally irradiated testes can be considered as an alternative to busulfan treatment, however, despite adverse and lethal effects, busulfan is commonly used for preparing a recipient animal models before transplantation of spermatogonial stem cells. 

In conclusion, busulfan consumption for cancer therapy has long-term consequences including reduced fertility and sometimes sterility. We found that two doses of busulfan injection, with 21 days interval, produced an appropriate animal model of induced azoospermia with comparable stereological indices of seminiferous tubules 35 days after the last injection in Sprague-Dawley rat. This model could be used for stem cell transplantation that could be carried out in the future studies.
